# Effective Macroscopic Thermomechanical Characterization of Multilayer Circuit Laminates for Advanced Electronic Packaging

**DOI:** 10.3390/ma16237491

**Published:** 2023-12-03

**Authors:** Hsien-Chie Cheng, Wen-You Jhu

**Affiliations:** 1 Department of Aerospace and Systems Engineering, Feng Chia University, Taichung 407, Taiwan; p1136209@o365.fcu.edu.tw; 2 Ph.D. Program of Mechanical and Aeronautical Engineering, Feng Chia University, Taichung 407, Taiwan

**Keywords:** laminate substrate, effective modeling, subregion modeling, trace mapping and modeling, finite element analysis, warpage process simulation

## Abstract

Laminate substrates in advanced IC packages serve as not only the principal heat dissipation pathway but also the critical component governing the thermomechanical performance of advanced packaging technologies. A solid and profound grasp of their thermomechanical properties is of crucial importance to better understand IC packages’ thermomechanical behavior. This study attempts to introduce a subregion homogenization modeling framework for effectively and efficiently modeling and characterizing the equivalent thermomechanical behavior of large-scale and high-density laminate substrates comprising the non-uniform distribution and non-unidirectional orientation of tiny metal traces. This framework incorporates subregion modeling, trace mapping and modeling, and finite element analysis (FEA)-based effective modeling. In addition, the laminates are macroscopically described as elastic orthotropic or elastic anisotropic material. This framework is first validated with simple uniaxial tensile and thermomechanical test simulations, and the calculation results associated with these two effective material models are compared with each other, as well as with those of two existing mixture models, and direct the detailed FEA. This framework is further tested on the prediction of the process-induced warpage of a flip chip chip-scale package, and the results are compared against the measurement data and the results of the whole-domain modeling-based effective approach and two existing mixture models.

## 1. Introduction

To date, the semiconductor manufacturing industry has been seeking a more effective and feasible technology solution to bridge the gap between cost, performance, and functionality. One of the effective solutions is the transition from transistor scaling to system scaling and integration (system on chip, SoC). An SoC that integrates all the required circuitry and functions, such as analog, logic, memory, and passives, into a single chip can be an ideal alternative for system integration. However, this technology faces several challenges, including high cost, low manufacturing yield, great physical design, and system chip testing complexities arising from its multifarious and heterogeneous nature. These challenges have further driven the industry to shift from SoC to heterogeneous system integration and scaling with advanced packaging, such as system-in-package (SiP) and system-on-package (SoP). These heterogeneous integration (HI) packaging solutions enable the integration of advanced packages [1,2], chips/chiplets [3], passive devices, and functional electronic components from various manufacturers with diversified manufacturing processes into a single package or module. They offer compelling efficiency, durability, flexibility, increased function density, and a more reasonable development and design cost, as well as good electrical performance, yield improvement, intellectual property reusability, and even miniaturization.

Presently, SiP and SoP have been effectively fulfilled using various advanced packaging technologies in a two-dimensional (2D) planar, two-and-a-half dimensional (2.5D) [3,4], or three-dimensional (3D) IC packaging configuration [5], such as package-in-package (PiP) [4], package-on-package (PoP) [6,7] using flip chip chip-scale packaging (FCCSP) [8,9], and flip chip ball grid array (FCBGA) packaging [10], and 3D IC integration with through silicon vias (TSVs) silicon or glass interposers [3,4,11,12]. When fulfilling these HI packaging technologies, high-density interconnect (HDI) IC substrates and interposers play a crucial role in providing electrical connection between the IC chip and the printed circuit board (PCB) and mechanical support and heat dissipation for IC chips. In addition, they play a dominant and essential role in the thermomechanical behavior of the electronic assemblies, driven by the mismatch between temperature and the coefficient of thermal expansion (CTE) among the constituent materials. Presently, many studies have been carried out to assess the thermal performance, reliability, and warpage of various laminate substrate-based IC packages and assemblies (see, e.g., [1,2,3,4,7,9,10,13]). Normally, this can be achieved using direct detailed finite element analysis (DD-FEA). However, circuit laminates, such as HDI IC substrates, interposers, and even PCBs, are typically complex and heterogeneous in nature and composed of multimaterial and multiscale structures, including insulation and protection layers of several micrometers in thickness and width, high-density circuit layers with a line width and space on the micrometer scale, and numerous blind and buried microvias and microscopic through holes. Precisely characterizing the circuit laminates using DD-FEA would face enormous difficulties in modeling effort and computational demand. Hence, there is a critical need for a more simplified and effective approach.

The macroscopic modeling of a heterogeneous composite has been a matter of great interest in recent studies. In the literature, various effective approaches have been proposed and used in FEA. Among them, representative volume elements (RVEs) or unit cells that denote the micro-level structural details and the material behavior of a composite as a whole are broadly utilized. This effective approach has also been extensively applied in microelectronic packaging to model the macroscopic behavior of, for example, TSV chips [14,15,16,17], redistribution layers (RDLs) [18], and PCBs [19]. For example, Cheng et al. [15] proposed an effective method based on 3D elasticity and FEA (briefly termed the FEA-based effective method) for macroscopically approximating TSV chips for 3D IC integration as a macroscopic transversely isotropic material. Lee et al. [18] applied three different effective approaches, i.e., the linear rule of mixture (ROM) approach, the representative volume approach, and RVE-based FEA, to model the macroscopic thermomechanical behavior of the RDLs of an RDL-first fan-out panel level package, where the copper (Cu) trace pattern was assumed to be uniformly distributed across the RDLs, and thus considered as an RVE. Nevertheless, this approach is generally ineffective for a non-uniform and non-periodic medium, such as IC substrates, interposers, and PCBs, which possess high heterogeneity and anisotropy due to the uneven distribution and non-unidirectional orientation of metal circuit traces. As a result, it is practically impossible to extract a periodic and repeated unit cell from metal circuit planes.

To macroscopically and thermomechanically describe multimaterial and multiscale circuit laminates, the most common effective approach is the simple linear (inverse) ROM estimate [14,15,16,17,18,19], namely, the Voigt (constant strain) and Reuss (constant stress) approximations. This approach has been broadly applied in the thermal or thermomechanical characterization of RDLs [16], PCBs [20], IC substrates [21], and underfill/bump layers [22,23] in microelectronics packaging due to its simplicity and ease of implementation. Unfortunately, this approach mostly lacks accuracy because of its inability to handle the elastic heterogeneity and anisotropy of circuit laminates, which are essentially two of the leading root causes of thermally induced warpages and material failures in electronic packages. As indicated in [7], a correct estimation of the equivalent thermomechanical properties of metal circuit planes and so the IC substrate is particularly indispensable for warpage control of the flip chip package-on-package. Accordingly, a more robust and effective method is highly desirable. Hutapea et al. [24] theoretically and experimentally investigated the equivalent elastic properties of a multilayer IC substrate and its warpage in an isothermal condition. Their approach involved the identification of complex substrate features, discretization of the substrate, and modeling and computation of the equivalent elastic properties of the identified features using an FEA-based effective method, considering the Cu trace orientation and volume fraction. Though this investigation is reliable and comprehensive, it is quite complicated, tedious, and cost-ineffective to implement for real applications. Later, an efficient and robust approach based on the trace mapping and modeling (TMM) technique and the FEA-based effective method was introduced with great success to macroscopically model circuit laminates, such as IC substrates [7,25] or PCBs [26]. The TMM technique is responsible for mapping each of the metal circuit planes in a circuit laminate onto a prebuilt 3D regular and fine mesh. In addition to simplicity, efficiency, and cost-effectiveness, this approach is more capable of managing highly heterogeneous and anisotropic metal circuit planes, thereby allowing for a more accurate estimate of their macroscopic elastic behavior.

Directly conducting trace mapping and modeling on whole large-scale and high-density circuit laminates remains exceptionally technically challenging because it requires an enormous mesh size and a very large computational effort. Thus, this study aims to propose a subregion homogenization modeling framework for effectively and efficiently characterizing the macroscopic elastic behavior of large-scale and high-density circuit laminates with high structural and material heterogeneity. The laminates are macroscopically considered as an elastic orthotropic or an elastic anisotropic material. These two material assumptions are implemented in the proposed framework to respectively create effective elastic orthotropic and anisotropic material models. To validate this framework, a set of simple uniaxial tensile and thermomechanical test simulations are performed on a piece of single Cu circuit layer and three-layer laminate. The simulation results associated with these two effective material models are compared with each other as well as with those of two mixture models and DD-FEA. The effectiveness of the proposed framework is further corroborated by the warpage simulation of an FCCSP package during fabrication.

## 2. Effective Modeling Approach

### 2.1. Rule of Mixture (ROM)/Analytical Estimate

**Figure 1:**
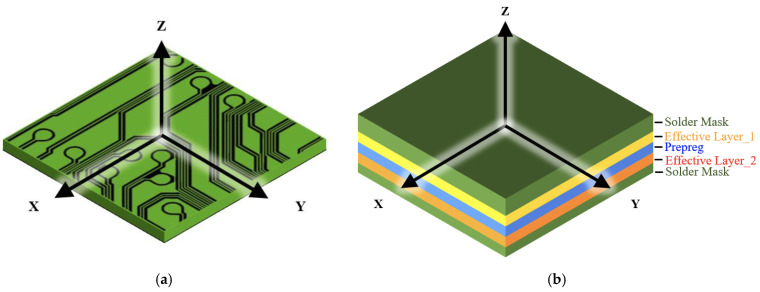
Schematics of a piece of (**a**) the metal trace layer and (**b**) circuit laminate.

A circuit laminate comprises a number of complex metal circuit planes. Figure 1 schematically depicts a piece of metal circuit plane and a circuit laminate. In the mechanics of materials, the classical Voigt (constant strain) and Reuss (constant stress) approximations are widely applied to explore the equivalent material properties of a multiphase composite, where the material components in this composite are connected in parallel and series, respectively. Basically, the Voigt model gives an approximation of the out-of-plane equivalent elastic modulus $E_{pc}^{eff}$ of parallel-connected materials, as shown in Figure 1b, as

(1) $$E_{pc}^{eff}=\sum_{i=1}^{n}E_{i}V_{i},$$

where *n* is the total number of material components in the composite and *E_i_* and *V_i_* are the Young’s modulus and volume fraction of the *i*th material component. The result obtained from Equation (1) happened to be the out-of-plane equivalent Young’s modulus for this particular metal circuit plane, as shown in Figure 1a.

The equivalent Poisson’s ratio $\nu^{eff}$ is approximated using the volume averaging (VA) technique, similar to Equation (1):

(2) $$\nu^{eff}=\sum_{i=1}^{n}\nu_{i}V_{i},$$

where $\nu_{i}$ is the Poisson’s ratio of the *i*th material component.

The Reuss model provides an estimate of the in-plane equivalent elastic modulus $E_{sc}^{eff}$ of series-connected materials, as also shown in Figure 1b, as

(3) $$E_{sc}^{eff}={\left(\sum_{i=1}^{n}\frac{V_{i}}{E_{i}}\right)}^{-1}.$$

Using the Voigt or Reuss approximation literally depends on the connection configuration (series or parallel type). As shown in Figure 1a, if the microscopic metal traces are not oriented in a unidirectional manner and distributed unevenly across the domain, the material properties are macroscopically approximated as an isotropic material using the VA technique.

On the other hand, the equivalent CTE of the series-connected ($\alpha_{sc}^{eff}$) and parallel-connected ($\alpha_{pc}^{eff}$) materials can be analytically estimated using, for example, an energy-based methodology [15,27] as follows:

(4) $$\alpha_{pc}^{eff}=\sum_{i=1}^{n}\alpha_{i}E_{i}V_{i}/\sum_{i=1}^{n}E_{i}V_{i},$$

(5) $$\alpha_{sc}^{eff}=\sum_{i=1}^{n}V_{i}\left(1+\nu_{i}\right)\alpha_{i}-\alpha_{pc}^{eff}\sum_{i=1}^{n}\nu_{i}V_{i},$$

where $\alpha_{i}$ is the CTE of the *i*th material component. It is worth nothing that the $\alpha_{pc}^{eff}$ also represents the out-of-plane equivalent CTE.

### 2.2. FEA-Based Effective Method

**Figure 2:**
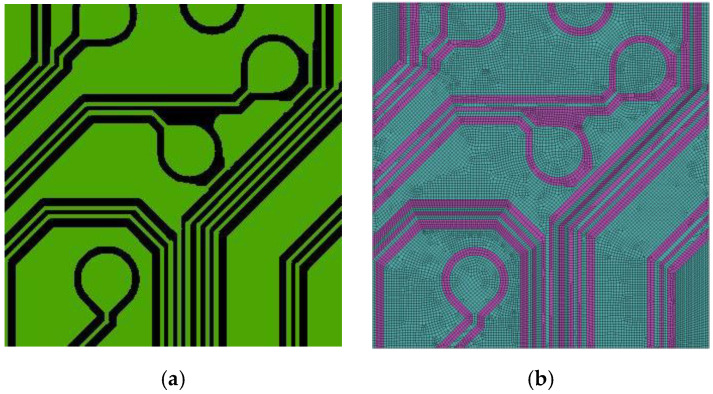
(**a**) Cu trace pattern. (**b**) Detailed finite element mesh.

The FEA-based effective method, as compared with the VA and linear (inverse) ROM techniques, is more effective and powerful for a precise estimate of the equivalent material properties of a composite, such as metal circuit planes (see, e.g., Figure 2), with a high degree of structural and material heterogeneity. The central principle of this approach is that the macroscopic elastic responses of the homogenized medium are elastically consistent with those of the original composite structure. The generalized Hooke’s law, defining the linear relationship between stress and strain, for an elastic orthotropic material can be rewritten as the following

(6) $$\varepsilon_{xx}=\frac{\sigma_{xx}}{E_{x}}-\frac{\nu_{yx}\sigma_{yy}}{E_{y}}-\frac{\nu_{zx}\sigma_{zz}}{E_{z}},$$

(7) $$\varepsilon_{yy}=\frac{\nu_{xy}\sigma_{xx}}{E_{x}}+\frac{\sigma_{yy}}{E_{y}}-\frac{\nu_{zy}\sigma_{zz}}{E_{z}},$$

(8) $$\varepsilon_{zz}=-\frac{\nu_{xz}\sigma_{xx}}{E_{x}}-\frac{\nu_{yz}\sigma_{yy}}{E_{y}}+\frac{\sigma_{zz}}{E_{z}},$$

(9) $$\gamma_{yz}=\frac{\tau_{yz}}{G_{yz}},$$

(10) $$\gamma_{xz}=\frac{\tau_{xz}}{G_{xz}},$$

(11) $$\gamma_{xy}=\frac{\tau_{xy}}{G_{xy}},$$

where $\varepsilon \left(\varepsilon_{xx},\varepsilon_{yy},\varepsilon_{zz}\right)$ and $\sigma \left(\sigma_{xx},\sigma_{yy},\sigma_{zz}\right)$ denote the normal strain and stress, respectively, $\gamma \left(\gamma_{yz},\gamma_{xz},\gamma_{xy}\right)$ and $\tau \left(\tau_{yz},\tau_{xz},\tau_{xy}\right)$ are the shear strain and stress, respectively, and $\nu \left(\nu_{yz},\nu_{xz},\nu_{xy}\right)$ represents Poisson’s ratio. In total, there are only nine independent elastic coefficients ($E_{x}$, $E_{y}$, $E_{z}$, $\nu_{yz}$, $\nu_{xz}$, $\nu_{xy}$, $G_{yz}$, $G_{xz}$, and $G_{xy}$) for the elastic orthotropic assumption.

For an elastic anisotropic material, the stress and strain relationship is expressed in matrix form as follows:

(12) $$\left\{\begin{array}{c} \sigma_{xx}\\ \sigma_{yy}\\ \sigma_{zz}\\ \tau_{yz}\\ \tau_{xz}\\ \tau_{xy} \end{array}\right\}=\left[c_{ij}\right]\left\{\begin{array}{c} \varepsilon_{xx}\\ \varepsilon_{yy}\\ \varepsilon_{zz}\\ \gamma_{yz}\\ \gamma_{xz}\\ \gamma_{xy} \end{array}\right\},$$

where

(13) $$\left[c_{ij}\right]=\left[\begin{array}{cccccc} c_{11} & & & & & \\ c_{21} & c_{22} & & & SYM & \\ c_{31} & c_{32} & c_{33} & & & \\ c_{41} & c_{42} & c_{43} & c_{44} & & \\ c_{51} & c_{52} & c_{53} & c_{54} & c_{55} & \\ c_{61} & c_{62} & c_{63} & c_{64} & c_{65} & c_{66} \end{array}\right].$$

In Equation (13), $c_{ij}$ is the elastic constant. Noticeably, in contrast with the elastic orthotropic assumption, there is an additional complicated relationship between the normal strains (stresses) and the shear stresses (strains) for the elastic anisotropic assumption. According to the principle of elastic strain energy, $\left[c_{ij}\right]$ is a symmetric matrix and thus comprises a total of 21 independent elastic constants.

The equivalent linear thermal expansion of a composite can be determined using the following expression:

(14) $$\Delta L_{i}=\alpha_{i}L_{i}\Delta T\;\left(i=x,y,z\right),$$

where $\Delta L_{i}$ stands for the thermal deformation, $\alpha_{i}$ denotes the equivalent CTE along the *i*th direction, $L_{i}$ is the side length of the composite medium in the *i*th direction, and ΔT represents the temperature increment. By virtue of a variety of thermal and mechanical loading conditions, these independent elastic constants can be derived simply using FEA.

### 2.3. The SFT Effective Approach

The macroscopic thermomechanical behavior of a large-scale and high-density circuit laminate when subject to thermomechanical loads can probably arise from the nonuniform distribution and non-unidirectional orientation of highly intricate, complex, and minuscule metal traces across the circuit planes, the dissimilarities in metal content among metal circuit planes, and the material heterogeneity in the laminate. Though the FEA-based effective method is a very effective and precise approach to estimating the macroscopic thermomechanical properties of a composite laminate, it requires an intensive effort in complicated geometry modeling, meshing, and computation, which makes it less practical, efficient, and feasible. These challenges can be well-released using the TMM technique. Although the TMM technique can be highly reliable, efficient, and effective for accurately modeling and macroscopically characterizing metal circuit planes, it still witnesses certain challenges in practical applications, particularly when metal circuit planes consist of a large dimension and very fine, intricate, complex and miniature metal traces.

**Figure 3:**
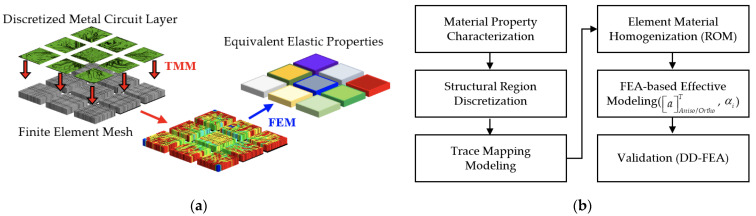
The proposed subregion homogenization modeling framework: (**a**) flowchart and (**b**) flow diagram.

**Figure 4:**
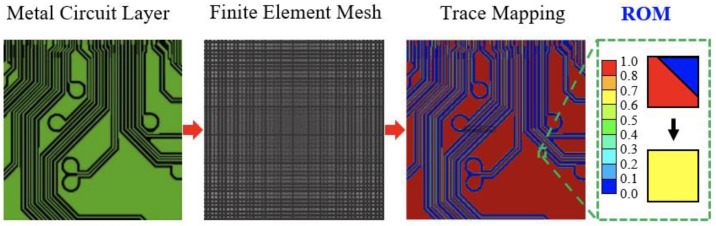
The TMM process.

To deal with this problem, a subregion homogenization modeling framework is introduced. This framework is developed by integrating the subregion modeling approach, the FEA-based effective method, and the TMM technique. A flowchart and flow diagram of this proposed framework are given in Figure 3. It is briefly termed the “SFT effective approach”. The procedure of this effective approach is illustrated as the following. Each of the metal circuit planes is first discretized into *N* pieces of small subregions. The size of these discretized subregions depends on the computational capability of computers, the degree of required accuracy, and the needed effort of modeling. In addition, the macroscopic material behavior of these subregions is assumed as a homogeneous and elastic medium, and considered to be orthotropic or anisotropic. Specifically, the modeling accuracy and effort will all increase with an increase in the number of subregions. Subsequently, the TMM technique is performed for each subregion of the metal circuit plane, as shown in Figure 4. First of all, a highly regular solid hexahedral finite element mesh is constructed, and the corresponding metal circuit layout in electronic computer-aided design (ECAD) is mapped onto it. Noteworthy is that to truthfully depict the complex, delicate, and intricate metal traces, the finite element mesh should be sufficiently fine while maintaining computational feasibility and cost-effectiveness. Some of the finite elements in the finite element mesh may be composed of two materials: one is the metal trace and the other is the dielectric insulation material or the protection material. Their elastic properties are assessed using the VA technique based on their volume fraction. Once the equivalent elastic properties of all these finite elements are derived, an elastic property map of the subregion is built, and the corresponding equivalent elastic properties are estimated using the FEA-based effective method. Similarly, an identical procedure is repeated for the other subregions until the elastic property map and the finite element model (FEM) of the entire metal circuit plane are established. The above subregion homogenization modeling procedure is also repeated for the other metal circuit planes. If further simplification is needed, the entire metal circuit plane constituted by the N subregions can be further treated as a homogenized elastic medium in which its equivalent elastic orthotropic or anisotropic properties can be explored using the FEA-based effective method. By assembling these metal circuit layers together with the dielectric insulation and protection layers, a 3D FEM of the entire circuit laminate is developed.

## 3. Results and Discussion

### 3.1. Validation of the TMM Technique

**Figure 5:**
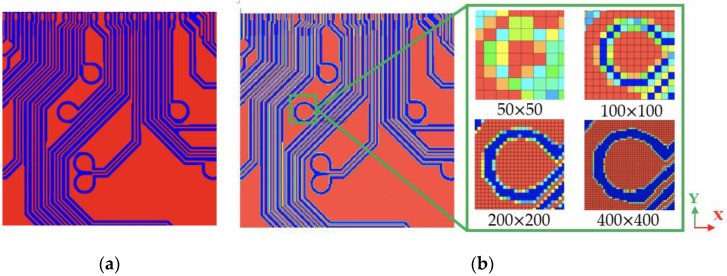
Construction of FEM for a piece of Cu circuit layer using: (**a**) direct detailed finite element modeling and (**b**) TMM.

To prove the feasibility of the TMM technique, a piece of Cu circuit layer, as shown in Figure 5, is utilized. The FEM of this piece of Cu circuit layer is constructed using DD-FEA and the TMM technique. As mentioned in the preceding section, in order to extract the structural and material details of the Cu circuit traces using the TMM technique, the pre-constructed regular finite element mesh needs to be sufficiently fine. For the tiny area circled by the square in the circuit layer in Figure 5b, four different mesh models are established for comparison, namely, 50 × 50, 100 × 100, 200 × 200, and 400 × 400. It is clear that a finer mesh leads to a higher resolution description of the geometric details of the Cu circuit traces. Undoubtedly, the 400 × 400 mesh model shows the best consistency with the direct detailed finite element modeling among these four mesh models, suggesting that the TMM technique can not only efficiently but also effectively model the complicated, intricate, and minuscule metal circuit traces if the grid size is small enough.

### 3.2. Validation of the Proposed Effective Approach

**Figure 6:**
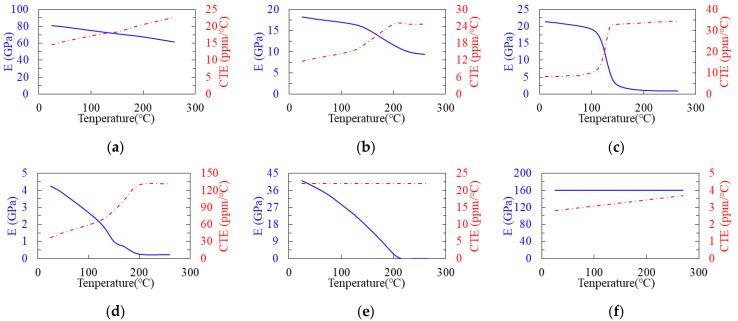
Temperature-dependent Young’s modulus and CTE of the materials: (**a**) Cu, (**b**) PP, (**c**) EMC, (**d**) SM, (**e**) solder (SAC305), and (**f**) Si chip.

The proposed SFT effective approach is first tested on a piece of a single Cu circuit layer under uniaxial tensile and thermomechanical loading conditions, and the results are compared against the DD-FEA and VA results. Note that the thermomechanical FEA is performed using ANSYS Workbench Mechanical (ANSYS, Canonsburg, PA, USA). The size of this single circuit layer is 2.0 (width) × 2.0 (length) × 0.022 (thickness) (mm). The Cu circuit traces enclosed with a prepreg (PP) material comprise a volume fraction of about 0.63. This single-circuit layer is macroscopically modeled as an elastic orthotropic or an elastic anisotropic material. The elastic properties of the materials in this single-circuit layer are shown in Figure 6, which are assumed to be linearly elastic and temperature-dependent. With the temperature-dependent elastic properties of these materials, the equivalent elastic orthotropic (${\left[a\right]}_{Ortho}$) and anisotropic (${\left[a\right]}_{\begin{array}{l} Aniso \end{array}}$) compliance matrices of this single-circuit layer are estimated using the proposed SFT effective approach at a different temperature, and the calculated results at 25 °C are shown below:

$${\left[a\right]}_{Ortho}^{T}=\left[\begin{array}{cccccc} 24.3 & & & & & \\ -6.7 & 21.1 & & & SYM & \\ -6.8 & -6.3 & 18.1 & & & \\ 0 & 0 & 0 & 61.9 & & \\ 0 & 0 & 0 & 0 & 49.5 & \\ 0 & 0 & 0 & 0 & 0 & 58.0 \end{array}\right]\times 10^{-6}$$

$${\left[a\right]}_{Aniso}^{T}=\left[\begin{array}{cccccc} 24.7 & & & & & \\ -6.1 & 21.3 & & & SYM & \\ -2.9 & -2.6 & 18.1 & & & \\ -0.1 & -0.2 & 0.6 & 61.9 & & \\ 0.0 & 0.0 & -0.2 & 0.0 & 49.5 & \\ 0.0 & 0.0 & -0.3 & 0.0 & -3.7 & 58.0 \end{array}\right]\times 10^{-6}$$

From these two elastic compliance matrices, this single-circuit layer reveals a more noticeable difference in the in-plane (X-Y) and out-of-plane (Z) equivalent elastic behaviors (i.e., a_11_/a_22_ vs. a_33_; a_44_/a_55_ vs. a_66_). Even in the in-plane properties (a_11_ vs. a_22_; a_44_ vs. a_55_), a minor discrepancy can also be observed. Furthermore, the calculated elastic anisotropic compliance properties show that the coefficient values: a_41_, a_42_, a_43_, a_51_, a_52_, a_53_, a_54_, a_61_, a_62_, a_63_, a_64_, and a_65_ are trivial, as compared with the diagonal terms, suggesting that this single circuit layer strongly exhibits orthotropic behavior. When this single-circuit layer is assumed as a homogeneous isotropic medium, the corresponding equivalent Young’s modulus, Poisson ratio, and CTE obtained using the VA technique are around 56.9 GPa, 0.32, and 10.9 ppm/°C at 25 °C.

With these calculated macroscopic material models, i.e., isotropic, orthotropic, and anisotropic, the elongations of this single-circuit layer in the X-, Y-, and Z-directions (U_x_, U_y_, and U_z_) subjected to various uniaxial tensile loading conditions, and a temperature swing of 100 °C is examined using FEA, and the results are listed in Table 1. For comparison, a DD-FEA that takes into account the structural and material details of the complex Cu circuit layer is also carried out. The differences in the results between the SFT effective approaches and the DD-FEA are also presented in the table. The table shows that in comparison with the VA technique, the results calculated using the proposed SFT effective approach, irrespective of whether it is an elastic orthotropic or anisotropic material assumption, agree much better with the DD-FEA results. This can be mostly attributable to the heterogeneity and anisotropy of the circuit substrate resulting from the non-uniform distribution and non-unidirectional orientation of Cu traces, and this is why the VA technique is unable to accurately predict the thermomechanical behavior of this heterogeneous composite lamina. Moreover, it is clear that the effective anisotropic model tends to give a more consistent result with the DD-FEA than the effective orthotropic model, but their discrepancy is minor. This result can be justified by the unimportant distinction in the predicted equivalent elastic orthotropic and anisotropic compliance matrices shown previously. This also suggests that the relatively simpler effective orthotropic model can be used in place of the more complex effective anisotropic model for estimating the macroscopic elastic behavior of the single-Cu circuit layer.

**Figure 7:**
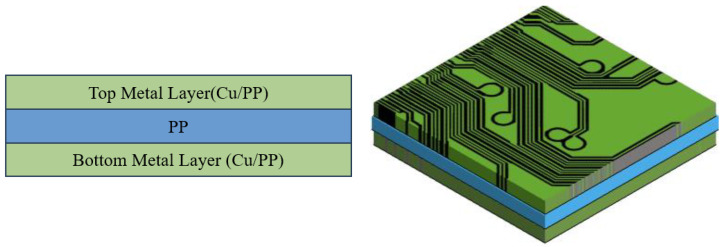
Schematics of a piece of three-layer laminate.

The efficacy of the proposed effective approach is additionally demonstrated with the aforementioned simple uniaxial tensile and thermomechanical test simulations on a piece of three-layer laminate, as shown in Figure 7. This laminate is made of one top circuit layer, shown in Figure 5, one PP middle layer with a size of 2.0 (width) × 2.0 (length) × 0.084 (thickness) (mm), and one bottom circuit layer with a size of 2.0 (width) × 2.0 (length) × 0.019 (thickness) (mm) and has a Cu volume fraction of about 0.81. Note that the size and Cu volume fraction of the top circuit layer were mentioned earlier. The predicted equivalent elastic orthotropic (${\left[a\right]}_{Ortho}^{B}$) and anisotropic (${\left[a\right]}_{Aniso}^{B}$) compliance matrices of the bottom Cu circuit layer using the proposed effective approach at 25 °C are presented as

$${\left[a\right]}_{Ortho}^{B}=\left[\begin{array}{cccccc} 17.3 & & & & & \\ -5.6 & 18.1 & & & SYM & \\ -5.4 & -5.6 & 14.8 & & & \\ 0 & 0 & 0 & 47.1 & & \\ 0 & 0 & 0 & 0 & 43.0 & \\ 0 & 0 & 0 & 0 & 0 & 41.7 \end{array}\right]\times 10^{-6}$$

$${\left[a\right]}_{Aniso}^{B}=\left[\begin{array}{cccccc} 17.4 & & & & & \\ -5.6 & 18.0 & & & SYM & \\ -5.5 & -5.4 & 14.8 & & & \\ 0.3 & 0.4 & -0.1 & 47.1 & & \\ 0.0 & 0.0 & 0.0 & 0.0 & 43.0 & \\ 0.0 & 0.0 & 0.0 & 0.0 & -1.5 & 41.7 \end{array}\right]\times 10^{-6}$$

The equivalent elastic isotropic properties (Young’s modulus, Poisson ratio, and CTE) of the bottom Cu circuit layer calculated using the VA technique at 25 °C are around 68.0 GPa, 0.33, and 12.7 ppm/°C. Furthermore, the equivalent elastic Young’s modulus and Poisson ratio of the entire three-layer laminate in the in-plane and out-of-plane directions are, respectively, calculated using the Voigt and Reuss models. For an elastic isotropic material assumption, the calculated in-plane and out-of-plane data using the Voigt and Reuss models are averaged, and the average Young’s modulus and Poisson ratio at 25 °C are 41.5 GPa and 0.31. The corresponding in-plane and out-of-plane equivalent CTEs are assessed using Equations (4) and (5) based on the energy-based approach [15,27], and they are 11.0 ppm/°C and 9.0 ppm/°C. This approach is hereinafter called the RV-average effective approach. Unlike the other approaches, i.e., VA and SFT, this approach conducts a second macroscopic constitutive modeling of this three-layer laminate by simply approximating the entire laminate as a homogeneous isotropic medium.

For each material model, a FEM is constructed, and its displacements under different uniaxial loads and a temperature load of 100 °C are calculated using FEA. Table 2 displays the calculated displacements and their differences from the DD-FEA results in percentage. It is not difficult to identify that a similar result was obtained in the case of the single-Cu circuit layer case, i.e., the effective elastic orthotropic and anisotropic models all significantly outperform the VA technique and the RV-average approach. In addition, these two effective models give a comparable result even though the effective anisotropic model slightly surpasses the effective orthotropic model. This implies that the effective orthotropic model could be a more preferred choice than the effective anisotropic one due to its relative simplicity and cost-effectiveness in determining the elastic compliance matrix (9 vs. 21). Furthermore, as compared with the VA technique, the RV-average effective approach results in a better estimate.

### 3.3. Validation Test on the Process-Induced Warpage Prediction of FCCSP

**Figure 8:**
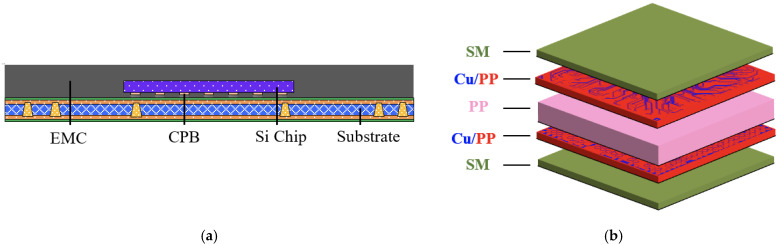
Schematics of (**a**) a cross-sectional view of an FCCSP package and (**b**) the coreless laminate substrate.

The applicability of the proposed SFT effective approach was further tested on the simulation of the process-induced warpage of an FCCSP package. In recent years, FCCSP, as schematically shown in Figure 8a, has been recognized as one of the leading packaging technologies because of its ability to achieve high I/O count, size reduction, and superior electrical characteristics. As a result, this packaging technology is a promising and encouraging solution to accomplish heterogeneous system integration. However, it still faces several challenges during fabrication. For example, the misalignment, mishandling, and misregistration caused by the process-induced warpage would strongly affect the process yield and throughput. Hence, it is crucial and imperative to comprehend the package’s warpage behavior during fabrication in the initial design phase.

In this section, the process-induced warpage behavior of the FCCSP is investigated using process simulation combined with several effective modeling approaches. This package mainly comprises a silicon (Si) chip, an epoxy molding compound (EMC), copper pillar bumps (CPBs), and a coreless laminate substrate. This laminate substrate (Figure 8b) is composed of two 13 μm thick solder mask (SM) layers at the bottom and the top, one 84 μm thick PP dielectric layer in the middle, and two Cu circuit layers, i.e., a 22 μm thick top circuit layer and a 19 μm thick bottom circuit layer. The Si die size is 4.15 (width) × 3.71 (length) × 0.193 (thickness) (mm). The chip is connected to the coreless laminate substrate using CPBs with a diameter of 90 μm and a height of 50 μm. The gap between the Si chip and the laminate substrate is filled with an EMC. The thickness of the EMC overmold above the Si chip is 220 μm. The size of the FCCSP is 11.1 mm (width) × 11.1 mm (length) × 0.601 (thickness) (mm).

**Figure 9:**
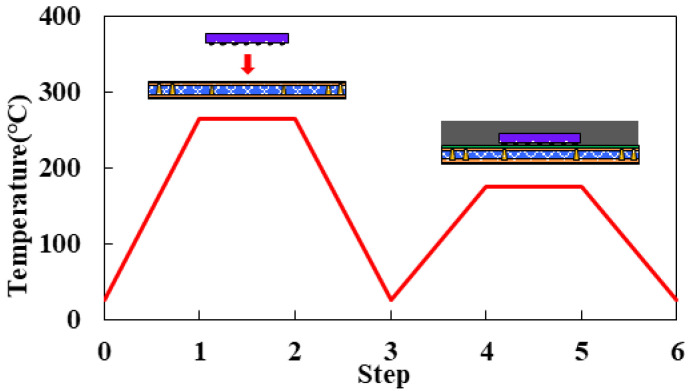
Fabrication process and process temperatures.

**Figure 10:**
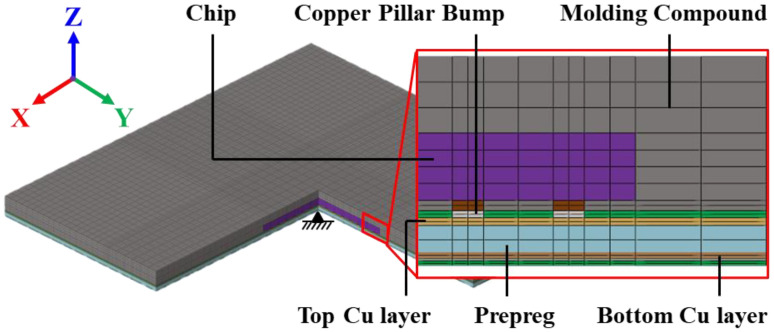
A 3D FEM of the FCCSP.

The fabrication process and the corresponding process temperatures of the FCCSP are shown in Figure 9, primarily including the die bonding process step (Steps 1–3) and the mold cure process (Steps 3–6). The process simulation integrates thermomechanical FEA, the element death and birth scheme, and effective modeling. The 3D thermomechanical FEM of the FCCSP is built with 172,660 nodes and 160,512 hexahedral elements, as shown in Figure 10. To avoid rigid body motion, four nodes near the center of the bottom plane of the laminate substrate are fully constrained. The materials in the FCCSP are all assumed linearly elastic and temperature-dependent, and their elastic properties characterized using a thermal-mechanical analyzer (TMA) and a dynamic mechanical analyzer (DMA) are displayed in Figure 6. The stress-free temperature is set at 175 °C.

In the proposed subregion modeling-based (i.e., SFT) and the whole-domain modeling-based (i.e., WFT) effective approaches, three different material models are considered for the coreless laminate substrate, namely, isotropic, orthotropic, and anisotropic. For the SFT effective approach, these two Cu circuit layers are individually divided into 3 × 3 subregions. It is noted that the WFT also uses the FEA-based effective approach to determine the temperature-dependent equivalent elastic properties of the entire Cu circuit layers. For the subregion-based VA (SVA) effective approach, i.e., “SVA (Isotropic)” in Table 3, the temperature-dependent equivalent elastic isotropic properties of each subregion are assessed using the VA technique based on the volume fraction of the Cu traces to the PP. These effective subregions, each of which consists of a different temperature-dependent equivalent elastic isotropic property, are further assembled to form an “inhomogeneous” IC substrate domain despite each subregion being considered a homogenous isotropic medium. On the other hand, for the whole domain-based VA effective approach, i.e., “WVA (Isotropic)” in Table 3, the temperature-dependent macroscopic elastic isotropic behavior of the entire laminate substrate is determined simply using the VA technique based on the volume ratio of the Cu traces to the other materials in the substrate. The calculated results using these effective models are compared with each other, and most importantly, with the experimental data.

**Figure 11:**
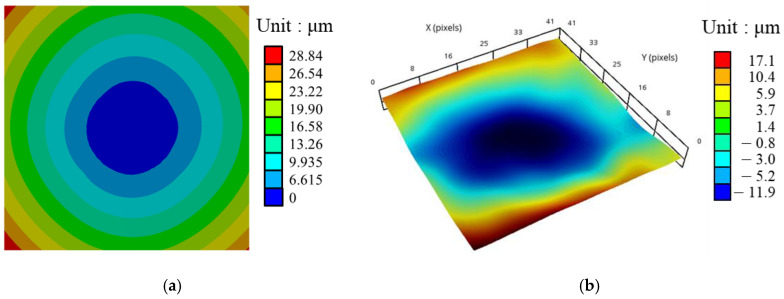
Warpage contour plots: (**a**) simulation (SFT with the anisotropic material model) and (**b**) measurement.

The simulated and experimental results of the FCCSP after the mold cure process are given in Table 3. The corresponding measured and simulated warpage contour plots are shown in Figure 11, where the simulated result is derived using process simulation based on the anisotropic-based SFT effective approach, i.e., “SFT (Anisotropic)” in Table 3. Both these two results demonstrate that the FCCSP would deform in a convex shape with a certain degree of asymmetry. This asymmetric warpage result would not be observed in homogeneous isotropic modeling, i.e., “WVA(Isotropic)”. The measured warpages are in the range of 18.0–31.0 µm with a mean value of 25.6 µm. Apparently, there is a very good match in the warpage results obtained with the measurement and the simulation that uses the SFT and WFT effective approaches. The difference between them is only about 9–12% for the effective elastic anisotropic model and 16–20% for the elastic orthotropic model. In addition to the warpage results, these two warpage contour plots are in good agreement with each other. Apart from that, the measured warpage profile exhibits a fairly greater degree of asymmetry than the simulated one. This could be caused by the structural and material heterogeneity in the circuit substrate as a result of the non-uniform distribution and non-unidirectional orientation of Cu traces, the thickness variation in the EMC, and uneven EMC curing, thereby likely resulting in variation in thermomechanical properties and nonuniform volume shrinkage [28]. Furthermore, according to the simulation results, it is found that the “SVA (Isotropic)” effective approach gives a slightly more consistent result with the experimental data than the “WVA (Isotropic)” approach. This result supports one’s physical intuition, and the tradeoff is the increased modeling effort and computational cost. In addition, it is also observed that the WFT effective approach would marginally surpass the SFT in the warpage prediction of the FCCSP under the same grid size, regardless of the material models. Despite that, the differences between them are quite insignificant, indicating that the SFT approach possesses greater modeling and computational efficiency while still retaining good prediction accuracy. However, when dealing with large-scale and high-density circuit laminates with highly intricate and inhomogeneous metal circuit layouts, the SFT approach would be a preferred and feasible choice. By further comparing the simulation results between the elastic orthotropic and anisotropic material models, one can easily observe that the anisotropic material model performs somewhat better than the orthotropic one. However, from the aspect of modeling effort and computational demand, the orthotropic material model is more attractive. Finally, it turns out that both the SFT and WFT effective approaches can provide a better prediction result than the WVA and SVA approaches.

## 4. Conclusions

A subregion homogenization modeling framework was presented for macroscopic modeling and characterization of the equivalent elastic properties of large-scale and high-density circuit laminates with high structural and material heterogeneity. The proposed framework (i.e., the SFT effective approach) is based on the integration of the subregion modeling approach, the FEA-based effective method, and the TMM technique. In this framework, the laminates are macroscopically modeled as an elastic orthotropic or elastic anisotropic material. The proposed framework was first tested on a piece of single-Cu circuit layer and three-layer laminate under uniaxial tensile and thermomechanical loading conditions. The applicability and robustness of the proposed framework were further verified on the warpage simulation of the FCCSP during fabrication. Below are some essential concluding remarks drawn from this research:

1. The TMM technique proves to be an easy, efficient, and robust approach for modeling highly complicated and intricate metal circuit layouts. In addition, a more refined mesh gives a higher-resolution description of the structural and material details of the Cu circuit traces.
2. The proposed SFT effective approach turns out to be an effective, efficient, and cost-effective tool for characterizing the equivalent elastic orthotropic and anisotropic properties of large-scale, high-density, multimaterial, and multiscale laminate substrates.
3. Irrespective of the use of an elastic orthotropic or anisotropic material model for the laminate substrates, the proposed SFT effective approach is shown to outperform the conventional mixture models (VA and RV-average) in macroscopically capturing the thermomechanical properties of the circuit laminates.
4. As compared with the WFT effective approach, the proposed SFT approach has superior efficiency and feasibility, especially in the macroscopic modeling of large-scale and high-density circuit laminates while still maintaining comparable prediction accuracy.
5. Though the effective elastic orthotropic model for the laminate substrates is slightly inferior in prediction accuracy to the effective elastic anisotropic one, its higher efficiency and cost-effectiveness make it the ideal and preferred choice for this problem.
6. The proposed SFT effective approach gives a better prediction of the process-induced warpage of the FCCSP, as compared with the WVA and SVA approaches.
7. The close agreement of the measured and simulated warpage results of the FCCSP after the mold cure process suggests the effectiveness of the proposed process simulation and the proposed subregion homogenization modeling framework.

## Figures and Tables

**Table 1:** Calculated displacements of the single-Cu circuit layer under uniaxial tensile and thermomechanical test simulations.

**Tensile Test**	**Ux (μm)**	**Diff. (%)**	**Uy (μm)**	**Diff. (%)**	**Uz (μm)**	**Diff. (%)**
DD-FEA	6.08	-	5.16	-	3.34	-
VA	4.19	31.1	4.19	18.8	3.26	2.4
SFT(Orthotropic)	5.79	4.7	5.03	2.4	3.37	0.7
SFT(Anisotropic)	5.88	3.3	5.06	1.8	3.41	2.0
**Thermomechanical Test**	**Ux (μm)**	**Diff. (%)**	**Uy (μm)**	**Diff. (%)**	**Uz (μm)**	**Diff. (%)**
DD-FEA	2.15	-	2.49	-	2.48	-
VA	2.17	0.8	2.17	12.8	2.28	7.9
SFT(Orthotropic)	2.16	0.2	2.43	2.4	2.41	2.5
SFT(Anisotropic)	2.16	0.2	2.43	2.4	2.41	2.5

**Table 2:** Calculated displacements of the three-layer laminate under uniaxial tensile and thermomechanical test simulations.

**Tensile Test**	**Ux (μm)**	**Diff. (%)**	**Uy (μm)**	**Diff. (%)**	**Uz (μm)**	**Diff. (%)**
DD-FEA	2.16	-	1.93	-	1.86	-
VA	1.67	22.6	1.67	13.4	1.18	36.4
RV-average	1.94	10.2	1.94	0.5	1.37	26.0
SFT (Orthotropic)	2.30	6.2	2.01	3.9	1.73	6.9
SFT (Anisotropic)	2.30	6.2	2.00	3.3	1.90	2.3
**Heating Test**	**Ux (μm)**	**Diff. (%)**	**Uy (μm)**	**Diff. (%)**	**Uz (μm)**	**Diff. (%)**
DD-FEA	2.17	-	2.41	-	52.79	-
VA	1.88	13.5	1.88	21.9	58.28	10.4
RV-average	2.19	0.8	2.19	9.0	55.76	5.6
SFT (Orthotropic)	2.00	7.8	2.29	4.7	52.72	0.1
SFT (Anisotropic)	2.02	6.9	2.30	4.5	52.92	0.2

**Table 3:** The experimental and simulated process-induced warpages of the FCCSP.

	Warpage (µm)	Difference (%)
Measurement	25.6 (18.0~31.0)	-
WFT (Anisotropic)	27.8	8.8
WFT (Orthotropic)	29.9	16.8
WVA (Isotropic)	36.9	44.3
SFT (Anisotropic)	28.8	12.8
SFT (Orthotropic)	30.8	20.3
SVA (Isotropic)	35.6	39.1

## Data Availability

Data are contained within the article.
